# The short board effect of ESG rating and corporate green innovation activities

**DOI:** 10.1371/journal.pone.0299795

**Published:** 2024-03-19

**Authors:** Fuxian Zhu, Xiaoli Xu, Jiachang Sun

**Affiliations:** 1 Department of Economics and Management, Xinjiang University, Urumqi, Xinjiang, China; 2 Department of Computer Science, Xinjiang University, Urumqi, Xinjiang, China; Inner Mongolia University, CHINA

## Abstract

This article aims to investigate whether differences in ESG ratings have an impact on corporate green innovation behavior. A high-order fixed effects model was established using panel data from Chinese companies from 2009 to 2022 to empirically test the impact of ESG rating divergence in the Chinese market on corporate green innovation behavior.The study demonstrates that ESG rating disparity raises the quantity but lowers the quality of businesses’ green innovation efforts because of the short board effect. After a series of robustness tests, the results are still valid.The mechanism investigation reveals that both an external pressure channel and an internal strategy adjustment channel are responsible for the impact of ESG rating disparity on green innovation efforts. The asymmetry of corporate green innovation activities is exacerbated by managers’ self-interest, whereas the asymmetry of green innovation is mitigated by the caliber of government. According to the heterogeneity analysis, the divergence of a business’s ESG rating between large-scale, non-heavy polluting, and places with strong environmental regulations can effectively slow down the asymmetric behavior of enterprise innovation activities. Additional investigation reveals that the phenomenon of ESG rating divergence spreads across industries and geographical areas. The short board effect of ESG rating divergence can be effectively mitigated by improving the quality of enterprise information disclosure and speeding up the digital transformation of businesses. The research conclusion provides marginal contributions on how to improve China’s ESG rating system and how enterprises can identify ESG rating differences and make scientific decisions.

## 1. Introduction

The notion of order between man and nature, as well as between man and society, is emphasized in traditional Chinese Confucianism. Ideas like "integration of nature and man," "taking in moderation and using in moderation," and "kindness to the people and love things" are examples of traditional wisdom that reflects our nation’s goal of harmony between man and nature as well as between man and society [1]. The development of the contemporary business system has given firms access to the moral importance of environmental,social and governance issues [2]. The term "ESG investment," also known as "sustainable investment" or "ethical investment," was first defined in 2006 by the United Nations-established Principles for Responsible Investment Organization. This definition includes the incorporation of environmental, social, and corporate governance considerations into investment decisions and shareholder investment strategies. Since then, the largest institutional investors in the world have rapidly committed to using ESG data when making investment decisions.According to the Sustainable Finance and ESG Development Report released by Reinitiv, since the establishment of the United Nations 2030 Agenda for Sustainable Development and the Paris Agreement in 2015, international regulatory policies related to ESG have doubled.However, in recent years, China’s popularity in the ESG field has been high, and ESG information disclosure has shown an optimistic trend, with many listed companies starting to issue ESG reports. As of June 2023, 1714 A-share listed companies have disclosed their social responsibility or ESG reports, an increase of 49.7% compared to the beginning of the previous year, with a disclosure ratio of 40%. In 2015, the Hong Kong Stock Exchange upgraded the ESG information disclosure standard to semi mandatory disclosure, proposing regulations to comply with or interpret; On December 18, 2019, the Hong Kong Stock Exchange officially released the third version of the ESG Reporting Guidelines, which focus more on post disclosure management and governance; In addition, some semi mandatory disclosure indicators have been upgraded to mandatory disclosure indicators.Since then, ESG concepts have gradually become popular in China.

An essential action guide for the application and practice of ESG in China is also provided by the excellent development that is suggested in the Report of the 20th CPC National Congress. With the growing popularity of ESG, ESG rating firms have proliferated in the financial markets. The ESG rating market is expanding quickly, and this has led to a significant difference in the ratings outcomes. Academics primarily concentrate on the reasons for rating disparity and its effects on the economy. Firstly, there are gaps in the breadth of information gathered by different rating agencies, the scope of index selection, and weight factors. These factors directly contribute to the divergence of ESG ratings [3]. Secondly, there is a lack of international standards for managing ESG information disclosure. Third, ESG rating procedures are not regulated. Finally, rating methods are proprietary and opaque. Second, ESG rating agencies are for-profit organizations that offer data, research, ratings, and advisory services on corporate governance and environmental, social, and governance practices of listed firms to financial institutions. Rating agencies that enter the established ESG rating market later than others typically create standards that differ from those already in place and provide ratings that differ from the current ones. In order to increase earnings from their established market share, more financial institutions are drawn to base investment decisions on their own rating findings. This also causes the ESG rating outcomes to become more diverse. ESG rating divergence is influenced by a number of other factors besides the uneven rating standards and the profit-driven nature of ESG rating firms. ESG rating divergence was found to be positively connected with ESG disclosure of rated units by Christensen et al. [4], meaning that the more information, the higher the rating divergence. This is also confirmed by Lium [5], who thinks that more significant ESG rating variance will result from non-standardized quantitative disclosure, particularly from the disclosure of environmental and social issues.Regarding the study of the economic effects of ESG rating divergence, Dimson E [6] thinks that ESG ratings can assist investors in making informed investment decisions and that investors can choose companies and build investment portfolios based on ESG investment indices. The concept of ESG rating discrepancy was confirmed by Doron Avramovdeng [7] using data from six rating agencies. But according to Liu et al. [8], the presence of ESG divergence enhanced stock price synchronism, decreased the incremental effect of information, and raised the degree of information asymmetry between investors and listed businesses. The danger of deviating from an enterprise’s rating performance, which takes into account factors like corporate social responsibility, social reputation, and internal operation efficiency, is shown in the ESG rating divergence. Providers will demand a higher investment premium in exchange for taking on this risk, which will drive up the cost of debt financing for businesses [9]. According to Wang et al. [10], auditors would become more aware of ESG rating divergence and, as a matter of professional prudence, would divulge more important audit concerns in order to lower their own risks. Researchers like He Taiming et al. [11] contend that when there is a large degree of dispute about ESG ratings, businesses usually try to mitigate unfavorable issues such principal-agent conflict and investor sentiment by disclosing more information.

Green innovation is a crucial tool for putting the idea of "green development" into practice since it can coordinate environmental growth while simultaneously achieving economic gains. China is now home to the second-biggest green bond market globally. The cumulative amount of green loans from 21 significant Chinese banks reached US $1.69 trillion by the end of 2020, demonstrating the consistent and long-term advancement of Chinese businesses in their transition to becoming green and sustainable. Economic policy uncertainty leads to fluctuations in financial markets [12]. In order for businesses to accomplish high-quality transformation and sustainable development in an uncertain environment, green innovation is a crucial internal driving factor [13]. The term "green innovation" describes the novel concepts, offerings, procedures, and organizational structures created to address environmental contamination. There are two primary areas of concentration for the influencing variables of corporate green innovation. There are small-scale influencing elements, on the one hand. According to Liu Chang [14], digital transformation offers businesses a new chance to accomplish leapfrog development and green transformation. It also serves as a driving factor. One major component that is seen to be necessary for businesses to implement green innovation is digital transformation. According to Pang and Zhao [15], CEOs who have a greater understanding of environmental preservation are also more receptive to the market prospects presented by green innovation. As a result, they are more likely to employ the "dark green" innovation strategy, which encourages the production of sustainable value. According to Lu and Jiang’s [16] analysis of manager characteristics, managers’ exposure to green practices influences corporate strategies to prioritize sustainable development, which in turn influences corporate strategic investment in sustainable development. According to Qi et al. [17], team members’ viewpoints and information are more comprehensive the more diverse their tenure is within the top management team. Furthermore, some academics predict that corporate green innovation will also be impacted by supply chain pressure [18], environmental information disclosure [19], environmental assessment [20], cross-border mergers and acquisitions [21], organizational redundancy [22], and other aspects. However, Qi Huaijin and Liu Siqin [23] thought that the green finance pilot legislation increased the variety of funding options available to businesses for their green technology innovation initiatives, which in turn increased the businesses’ passion for green innovation. This was from a macro perspective. Yu Bo [24] came to the conclusion that green financing policies have contributed to the effectiveness and caliber of enterprise green innovation based on the Porter Effect theory. According to Li Ping and Fang Jian [25], environmental regulations will raise business expenses in the near run, but over time, businesses might reduce those costs by using innovative strategies that boost operating efficiency. Environmental regulations lower the uncertainty surrounding the value of businesses’ investments in the environmental sector, influence business expectations, and spur business innovation in this area [26]. Furthermore,Strategic behavior of enterprises will be impacted by the digital economy [27, 28], carbon pilot policy [29], public environmental attention [30], industrial agglomeration [31], and Chairman’s age [32].

The available literature offers a significant theoretical foundation for our inquiry, as indicated by the analysis above. But the majority of the research that has already been done on the subject focuses on corporate behavior and the effects of external policies and regulations on corporate green innovation activities. There is, however, a dearth of studies that examine corporate green innovation behavior from the standpoint of soft constraints, particularly the emerging field of ESG rating divergence, whose effects on corporate green innovation activities have not been examined as an external driving factor. This article examines the impact of ESG rating divergence on corporate green innovation efforts for three reasons, despite the fact that it may influence corporate behaviors from many angles. To begin with, green innovation and ESG ideas mesh really well. On the other hand, such green behaviors can also benefit upstream and downstream enterprises, society, employees and government departments, reflecting the S dimension. Consequently, in order to offer further research perspectives on the relationship between ESG rating and micro green innovation activities, corporate green innovation activities are chosen as the starting point for the investigation of the economic effects of ESG rating divergence.Based on this, this article focuses on the enterprise’s green innovation operations, builds the ESG rating disagreement index, analyzes experimentally whether soft regulation based on ESG rating disagreement may enhance the effectiveness of green innovation, and looks into the channel and mechanism of its influence.

**Fig 1:**
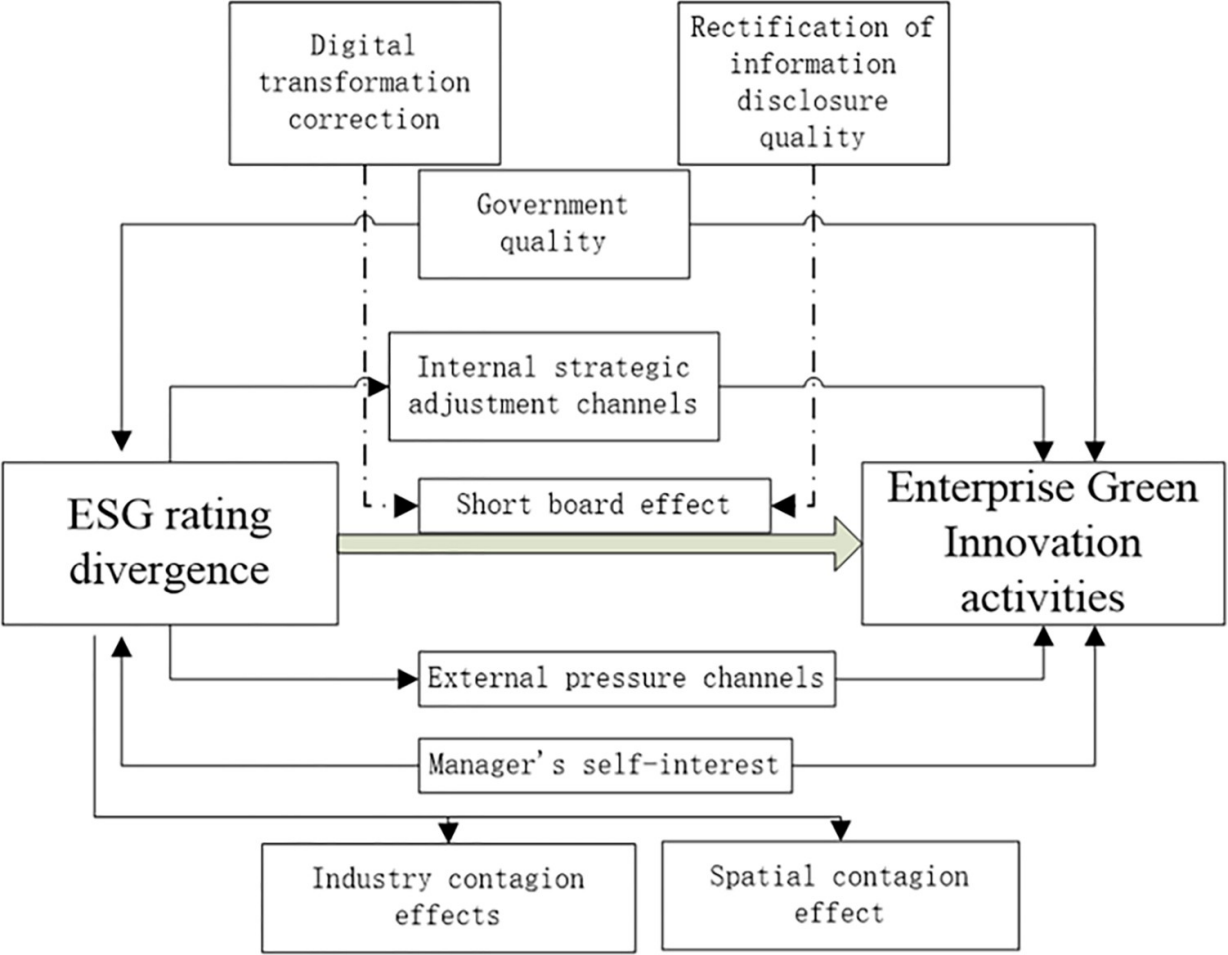
The impact of ESG rating divergence on green innovation activities of enterprises.

The following factors mostly show this paper’s modest contribution when compared to earlier research. First, based on the short board effect, this paper discusses the influence mechanism of ESG rating divergence on enterprise green innovation activities, and enriches the research perspective of the micro effect of ESG rating divergence.Second, this article finds that disagreements over ESG ratings will affect green innovation activities through two channels: internal strategy adjustment and relieving external pressure, based on the green innovation activities of micro companies. This demonstrates the interactive relationship between ESG rating divergence and the asymmetric phenomenon of green innovation, in addition to adding to the pertinent literature on informal limitations. Thirdly, the extent of enterprise pollution, the enterprise size, and the diverse effects of environmental regulatory intensity in the region where the enterprise is located are all thoroughly examined in this paper. Fourthly, the research presented above leads this paper to conclude that there is a contagion effect of ESG rating divergence across industries and regions. The short board effect of this phenomenon can be mitigated by improving the quality of enterprise information disclosure and expediting the digital transformation of businesses. Government departments can use this as a policy reference to further enhance the ESG information disclosure and evaluation systems. The workflow diagram of this article is shown in Fig 1.

The remaining part of this article is arranged as follows: Chapter 2 is the theoretical foundation and research hypotheses; Chapter 3 is variable selection and model design; Chapter 4 is empirical analysis; Chapter 5 is for further research; Chapter 6 is Conclusion and Suggestions.

## 2. Theoretical basis and research hypotheses

Famous American management scientist Lawrence Peter first proposed the extensive short-board effect in the 1960s. The main idea behind it is that, while the long board can estimate the barrel’s capacity, the shortest board really calculates the actual volume of water that can be held. Rather than its strongest or longest link, a system’s total performance is frequently constrained by its weakest or shortest link [33]. ESG rating is progressively incorporated into investors’ investment decisions as more and more financial institutions join the responsible investment movement. This leads to the formation of a hundred schools of disagreement in the ESG rating sector. While the rating discrepancy provides more information, its utility is diminished. Owing to the short board effect, profit-driven investors frequently choose businesses with overall high scores when making investments, forgoing those with significant rating volatility despite varying ESG rating agency findings. For instance, when running the two businesses Z and X under the same other circumstances, according to four ESG rating agencies, business Z receives a score of B, while three of the businesses X receive an A, and the final one receives a C. Even when enterprise Z has a lower average ESG score than enterprise X, investors nonetheless opt to invest in enterprise Z due to the short board effect and the desire to minimize risks. Similarly, the principal-agent relationship will likewise exhibit this short-board effect. The short-board effect, then, causes stakeholders to focus excessively on the low score, which has an impact on business operations and investor decisions.

Managers will alter their strategic choices to satisfy the mainstream requirements of an ESG grade in an effort to lessen shareholder accountability and draw in investors. Green innovation is a crucial component of the ESG rating standard because it can be used to boost operating efficiency and generate financial gains in addition to meeting the requirements of the rating agencies to receive higher scores [34, 35]. This will surely encourage businesses to implement green innovation in order to comply with the ESG rating standards.

Managers have more latitude in accommodating ESG ratings because of the inconsistent nature of ESG rating criteria and rating frequency [36]. First and foremost, there is a connection between corporate managers’ performance and ESG ratings. Owing to the principal-agent problem and managers’ myopia, they frequently select innovation projects that will yield immediate benefits or undertake a high number of low-level innovations in an effort to minimize public pressure and reap as many benefits as they can during their brief term of office [37]. they overlook innovation initiatives that have the potential to increase the long-term value of businesses. As a result, in the event of a significant discrepancy in ESG ratings, the management will come under intense pressure from the government, shareholders, and outside investors to increase its investment in innovation activities and to drive the company to undertake low-level innovation in order to satisfy the demands of innovation "quantity." This leads to greater differences in ESG ratings, resulting in more low-quality green innovation for enterprises. Second, conflict will lower the caliber of green innovation initiatives undertaken by businesses. Green patent citations have a lengthy time lag effect and are a significant predictor of the "quality" of green innovation produced by businesses [38]. The rationale is that, although patent citations can only demonstrate a cumulative effect in subsequent years, some ESG rating agencies only evaluate an organization’s innovation activity in the current year. In the current period, it is challenging to objectively and uniformly evaluate the quality of firms’ green innovation due to rating agencies’ disregard for time-lag issues, which leads to increasingly inconsistent outcomes. In addition to lessening the crowding-out effect caused by this portion of invested funds going toward other enterprise production activities, moving funds from high-quality innovation to the accumulation of innovation quantity at this time can also better meet mainstream ESG rating standards and lessen the degree of divergence. This leads to an asymmetric phenomenon where as the ESG divergence increases, there are more low-quality green innovations and fewer high-quality green innovations. Consequently, the ensuing presumptions are made:

First hypothesis: The divergence in ESG ratings will encourage more companies to engage in low-quality green innovation, while hindering green innovation in terms of quality.

For-profit organizations known as ESG rating agencies offer financial institutions data, research, and advisory services related to corporate governance and environmental, social, and governance (ESG) of listed firms. Investors and financial institutions utilize ESG rating information in addition to businesses. There are variations in the rating caliber of rating agencies alongside the rise in their number in the market [11]. Additionally, different stakeholders have varying preferences when it comes to the information that rating agencies choose to present. External investors, for example, frequently select institutions with low scores as the weak board of the "investment barrel," giving low scores more weight than good ones. Corporate managers, however, frequently choose to hide the poor scores and present a positive public image by only disclosing the ratings with higher scores. The principal will be on the lookout for the disagreement as an unfavorable indication, even if the management would like select a better score as the representation of sustainable development. Or do the principles we once adhered to have changed? Under such pressure, the management will review the development idea, review the corporate strategy, and give more consideration to green investment initiatives that align with the ESG score concept and add value to the business [8]. However, because of the divergence, there will be less trust in the veracity of the enterprise information, and external stakeholders will question whether the ESG information is overstated and whether the stock price spillover phenomenon is real. As a result, the enterprises will be forced to either change the investment object or pay a higher risk compensation, which will put a significant financial burden on the rated enterprises and create financing constraints. Disagreements over enterprise innovation initiatives through external channels resulting in an ESG rating have led to all of these bank runs [39].

The second hypothesis: There will be two ways in which the disagreement over ESG ratings will affect corporate green innovation activities: external pressure and internal strategic adjustment.

## 3. Variable selection and model design

### 3.1 Variable selection

#### (1) Explanatory and explained variables

*① Explanatory variable*: *ESG divergence*. In order to measure ESG rating divergence, six rating agencies including Bloomberg, Wind, FTSE Russell, China Securities, Syndao and Menglang are selected as the source data. Because the FTSE Russell score range is 0–3; China Securities and Bloomberg use the percentage system; The Wind score range is 0–10 points; The channel is scored according to nine grades: A+, A, A-, B+, B, B-, C+, C and C-, with 9–1 points respectively; Munang assigns a score of 1–27 to 27 on a scale that varies from agency to agency. Finally, referring to the practice of DoronAvramov [7] and Fang Xianming [40], for each enterprise, the uncertainty of paired rating is calculated, and 15 rating differences are formed by the six rating agencies. Finally, the inverse of the standard deviation of these 15 differences is calculated and the logarithm is taken to obtain the rating disagreement (disesg) of the enterprise in that year.The larger the value, the smaller the difference in ESG ratings for the company.

*②Explained variable*: *Green innovation*. Referring to the practice of Liu et al. [14], the logarithm of the number of green invention patent applications of enterprises in the current year plus 1 is used to measure the number of enterprises’ green innovation (Gptotal). Secondly, referring to the method of Lahiri [41], we collect, sort out and match the sum of the number of citations of green patents applied by enterprises in the current year in the next two years, and remove the number of citations of enterprises themselves, and then add 1 to take the natural logarithm to measure the quality of green innovation (Gpcitation).

#### (2) Mechanism variable

*①Cost of equity financing*. Referring to the research of Luo et al. [42], this paper uses PEG model to estimate the equity financing cost (Cec). This model can well capture all kinds of risks and consider the enterprise value and income growth factors, with high scientific and reliable. The specific calculation formula is as follows:

$$COE_{PEG}=\sqrt{\frac{EPS_{t+2}-EPS_{t+1}}{P_{t}}}$$

EPSt+1,EPSt+2 are the forecasted net earnings per share of the enterprise at the end of t+1 and t+2 respectively, and Pt is the closing price of the stock of the enterprise in period t.

*② Analyst attention*. Referring to the practice of Chen et al. [43], the natural logarithm (Follow) is taken after adding 1 to the total number of analysts following an enterprise in the research year to measure analyst attention (AAttention).

*③ Degree of media attention*. Referring to the practice of Du et al. [44], we measure the media attention (Newsnum) according to the logarithm of the number of media reports of listed companies in the current year plus 1. The data of media reports come from China Financial News Database of Listed Companies (CFND).

*④ Research and development efforts*. Referring to the practice of Song and Liu [45], the amount of R&D investment is used as the proxy variable of R&D intensity (RD).

*⑤ Speed of asset update*. Referring to the practice of Dai Xiang and Yang Shuang Zhi [46], we use the total amount of assets amortization and depreciation in the current year to quantify the Dep.

*⑥ Level of financialization*. Referring to the measurement method of Duchin et al. [47], the financialization of real enterprises = (trading financial assets + net financial assets available for sale + net investment real estate + net loans and advances + net held-to-maturity investments + derivative financial assets + financial assets in other current assets and long-term equity investments)/ total assets. The larger the value is, the higher the proportion of corporate financial assets in total assets is, and the higher the level of finratio is.

*⑦ Self-interest of managers*. Managers’ self-interest (overpay), referring to the practice of Wu Liansheng et al. [48], uses managers’ excess pay to measure managers’ self-interest. As explanatory variables, company size (size), financial leverage (lev), company performance (roa), integration of two positions (cbd), board size (bds), ultimate controller type (soe) and management shareholding ratio (son) are selected to construct a management compensation determination model and calculate the actual management compensation of each company. The above model, industry and year are controlled for regression, and the estimated coefficient is then put into the actual compensation model, and the residual is the excess compensation of the management in the current year (Overpay1).

*⑧ Quality of government*. Referring to the practice of Zhen Meilong and Jiang Xiaozhuang [49], the intellectual property protection index and the government scale reduction index in the China Marketization Index compiled by Fan Gang [50] are selected to measure the level of intellectual property protection and the level of maintaining normal market activities of each local government respectively, and then the average is calculated to measure the comprehensive government quality index (GQ) of each local government. A higher value indicates a higher quality of local government.

#### (3) Control variables

Based on the existing research, we select enterprise Size (Size), financial risk (RCE), enterprise nature (SOE), enterprise age (ListAge), enterprise Growth (Growth) and ownership concentration (Top5) as control variables. The variable definition is shown in Table 1.

**Table 1:** Variable definition table.

Variable type	Variable	Variable abbreviation	Variable Declaration
Explained variable	Green Innovation Quality	GPcitation	LN (The total number of citations of green patents applied for by enterprises in the next two years+1)
	Number of green innovations	GPtotal	LN (Number of green patent applications by enterprises in the current year+1)
Explanatory variable	ESG divergence	disesg	LN (1/Standard deviation of ESG ratings from various rating agencies)
Control variable	Enterprise size	Size	LN(Total fixed assets)
	Financial risk	RCE	Accounts receivable/operating income
	Enterprise nature	SOE	Take 1 for state-owned enterprises and 0 for non-state-owned enterprises
	Enterprise age	ListAge	Listing period
	Enterprise Growth	Growth	Operating revenue growth rate
	Ownership concentration	Top5	Shareholding ratio of the top five major shareholders
Mechanism variables	Equity financing costs	Cec	Calculated by PEG model
	Analyst attention	AAttention	LN (Total number of analysts tracking a company+1)
	Media attention	Newsnum	LN (Number of media reports from listed companies+1)
	R&D efforts	RD	R&D investment amount
	Asset update speed	Dep	(Asset amortization+depreciation for the year)/10^8^
	Financial level	finratio	Financial assets/total assets
	Manager’s self-interest	overpay	Measurement of excess compensation
	Government quality	GQ	(Intellectual property protection level+maintenance of normal market activity level)/2

### 3.2 Sample selection and model setting

Using the annual data of A-share listed companies from 2009 to 2022 as a sample, this study empirically tests the impact of ESG rating divergence on corporate green innovation behavior. The data sources are separated into three groups. First, the CSMAR database provides fundamental company information and financial indicators; Second, the official websites of each ESG rating agency provide the ESG rating data; and Third, the CNRDS database provides information on corporate green innovation patents. Second, the following pre-processing is done to the data: (1) The samples from the financial business are removed. (2) Removing samples marked as ST and ST*. (3) The sample becomes more representative when companies that routinely receive bad ratings from rating agencies due to their noncompliance with ESG scoring guidelines are excluded. For empirical analysis, the multi-dimensional fixed effect model controlling for individual, industry, and year is employed. The purpose of doing this is to eliminate the distortion of regression results caused by individual, industry, and annual differences. Thus obtaining the net effect of ESG rating divergence on corporate green innovation activities. The model has the following design:

$$Gpcitation_{it}=disesg_{it}+Size_{it}+REC_{it}+SOE_{it}+ListAge_{it}+Growth_{it}+Top5_{it}+\gamma +\lambda +\delta +\varepsilon_{it}$$ (1)

$$Gptotal_{it}=disesg_{it}+Size_{it}+REC_{it}+SOE_{it}+ListAge_{it}+Growth_{it}+Top5_{it}+\gamma +\lambda +\delta +\varepsilon_{it}$$ (2)

*Gpcitation*_*it*_ represents the quality of green innovation, *Gptotal*_*it*_ represents the quantity of green innovation, *disesg*_*it*_ represents the degree of disagreement, *Size*_*it*_、 *REC*_*it*_、*SOE*_*it*_、 *ListAge*_*it*_、 *Growth*_*it*_、*Top*5_*it*_ represents the control variable, and *γ*、*λ*、*δ* respectively represents the year, individual and industry fixed effects, and *ε*_*it*_ is the random disturbance term.

## 4. Empirical results

### 4.1 Descriptive statistics

Table 2 shows the descriptive statistics of the main variables.The peak value of green innovation(GPtotal) is 8.384, the valley value is 0, and the standard deviation is 1.161, indicating that there is still a significant difference in the number of green innovations among enterprises.The peak value of green innovation quality(GPcitation) is 8.355, the valley value is 0, the standard deviation is 1.207, and the average value is 0.689, indicating that the overall green innovation quality of the enterprise is low and the level of innovation ability is uneven.The minimum ESG rating divergence is 0.791, the maximum is 9.643, the average is 2.526, and the standard deviation is 0.785, indicating that there are significant differences in the rating results given by ESG rating agencies, and the phenomenon of rating divergence is widespread.

**Table 2:** Descriptive statistics.

Variable	N	Mean	SD	Min	Max
**GPtotal**	39920	0.529	1.161	0	8.384
**GPcitation**	39980	0.689	1.207	0	8.355
**disesg**	26712	2.526	0.785	0.791	9.643
**Size**	39980	22.131	1.321	14.942	28.509
**REC**	39875	0.121	0.105	0	0.813
**SOE**	39980	0.332	0.471	0	1
**ListAge**	39980	1.995	0.964	0	3.497
**Growth**	39958	0.705	75.267	-1.309	14883.060
**Top5**	39942	0.538	0.156	0.008	0.992
**Cec**	8776	0.086	0.201	-15.597	4.002
**AAttent**	5694	1.265	1.141	0	4.143
**newsnum**	40027	281.638	868.456	0	66342
**RD**	6896	5.307	1.267	0	13.636
**Dep**	35622	3.916	3.493	-5.262	2.194
**finratio**	35586	-0.167	14.493	-156.615	1922.998
**Overpay**	38186	0.001	0.550	-2.174	2.456
**GQ**	39561	7.679	1.630	-0.755	10.972

### 4.2 Benchmark regression

The benchmark regression’s findings are presented in the Table 3 that follows. Firstly, the first and second columns are univariate regression, and the results show that differences in ESG ratings can have a significant impact on corporate innovation activities.Second, the results indicate that disagreement hinders both the amount and quality of green innovation when control factors are added to the model in columns 2 and 3 without accounting for industry, person, or temporal effects. Ultimately, the addition of the fixed effect results in an asymmetric influence of ESG rating dispute on corporate green innovation activities. Specifically, the quantity of corporate green innovation will increase due to ESG rating disagreement, while the quality of corporate green innovation would decrease.

**Table 3:** Benchmark regression of the impact of ESG rating divergence (disesg) on green innovation in enterprises (GPtotal、GPcitation).

	(1)	(2)	(3)	(4)	(5)	(6)
	GPcitation	GPtotal	GPcitation	GPtotal	GPcitation	GPtotal
**disesg**	-0.0166***	0.0350***	-0.0651***	-0.0790***	-0.0216***	0.0327***
	(-2.7138)	(3.9751)	(-9.0544)	(-10.2821)	(-3.5201)	(3.6806)
**Size**			0.4719***	0.3582***	0.1884***	0.1972***
			(61.2348)	(45.0537)	(12.7679)	(10.7362)
**REC**			0.8893***	0.6361***	0.1012	0.8789***
			(10.9782)	(8.3405)	(0.9417)	(6.0458)
**SOE**			0.0662***	0.2087***	0.0638*	0.1762***
			(3.7947)	(11.9438)	(1.6950)	(3.7803)
**ListAge**			0.1703***	-0.0478***	0.2755***	0.1211***
			(19.2313)	(-5.2522)	(12.3564)	(4.6941)
**Growth**			-0.0000**	-0.0000**	0.0000***	0.0000
			(-2.2719)	(-1.9818)	(2.8961)	(0.5605)
**Top5**			-0.3056***	0.0625	-0.0611	0.0349
			(-6.1152)	(1.2800)	(-0.7244)	(0.3263)
**_cons**	-0.3514***	0.1659***	-9.9334***	-7.3867***	-4.7160***	-4.3731***
	(-7.1801)	(3.3211)	(-59.3389)	(-42.7120)	(-14.9121)	(-11.1067)
***N***	26429	26296	26699	26566	26344	26211
**r2_a**	0.8074	0.5319	0.3574	0.2232	0.8135	0.5371

Notes:This table presents fixed-effects estimation of the relationship between ESG rating divergence and green innovation in enterprises

***, ** and * indicate significance at the 1, 5 and 10% levels, respectively.

### 4.3 Robustness test

#### (1) Exclusivity test

This report suggests the following potential alternate hypotheses to guarantee the validity of the research findings: (1) Changing the methodology for calculating disagreement: in the benchmark regression, the samples that were removed because additional variables were not present in the entire rating enterprise sample are referred to by the standard deviation that was computed using the sample’s rating data. In the robustness, the size of the population is utilized to obtain the disagreement (disesg2), which is displayed in columns 1–2 of Table 4, and the disagreement samples of the population are used to compute the standard deviation of the average value. (2) Analyst forecast (FDISP), like ESG rating, is an impartial, impartial, and objective method of soft market regulation. This research examines the effect of industry-specific regulation on analyst forecast, drawing on the work of Zhou et al. [51]. Dispersion seen through each analyst’s unique lens. Greater convergence in analyst estimates is indicated by higher values. The outcomes are displayed in Table 4’s columns 3–4. (3) The explanatory variables are regressed with a lag of one period to see if there is reverse causality between corporate green innovation efforts and ESG rating discrepancy, as seen in Columns 5–6 of Table 4. (4) Leaving out the 2022 sample data. China’s economic progress was forced to slow down and stall in 2022, most notably because to the COVID-19 outbreak in 2020, and production and corporate operations faced challenges. Businesses are now seen as engaging in a unique state of production and management, in contrast to the previous paradigm that they adhered to. Therefore, the results are displayed in Table 4’s columns 7–8 in order to remove the interference caused by external shock occurrences. Green innovation activities do virtual variable (GPcitationdumm/GPcitation), (5) based on the quantity and quality of green innovation data characteristics, there will be no green innovation an application for a patent for invention and cited the enterprise from zero, there will be a green invention patents and patent cited times greater than 5 green enterprise take 1, The Probit model is used for the regression test, and Table 5’s columns 1–2 display the outcomes.

**Table 4:** Robustness testing based on variable replacement, reverse causality, and sample replacement.

	(1)	(2)	(3)	(4)	(5)	(6)	(7)	(8)
	GPcitation	GPtotal	GPcitation	GPtotal	GPcitation	GPtotal	GPcitation	GPtotal
**disesg2**	-0.0118**	0.0165*						
	(-1.9733)	(1.8071)						
**Size**	0.1244***	0.0704***	0.2431***	0.2163***	0.1862***	0.1762***	0.2158***	0.2807***
	(6.6175)	(2.9237)	(9.6585)	(7.7417)	(11.1059)	(7.9266)	(12.4289)	(13.2374)
**REC**	0.1360	0.9656***	0.3599*	1.0987***	0.0607	0.7863***	0.0408	0.8485***
	(1.2557)	(5.8566)	(1.7900)	(4.4046)	(0.4891)	(4.5476)	(0.3220)	(5.0308)
**SOE**	-0.0263	0.0713	0.0418	-0.0598	0.1007**	0.1894***	0.0924**	0.2061***
	(-0.7540)	(1.3760)	(0.5587)	(-0.6996)	(2.3322)	(3.5058)	(2.0922)	(3.8372)
**ListAge**	0.4125***	0.2362***	0.0687**	0.1243***	0.2943***	0.1882***	0.1630***	0.0666**
	(19.5937)	(7.6857)	(2.1272)	(3.2646)	(7.9187)	(4.0435)	(5.7656)	(2.1692)
**Growth**	-0.0003*	-0.0002	-0.0128**	-0.0061	-0.0005***	-0.0003**	0.0001***	0.0000
	(-1.9127)	(-1.1781)	(-2.0041)	(-1.5100)	(-3.1380)	(-2.1910)	(3.3775)	(1.3084)
**Top5**	0.0240	0.0419	-0.4193***	0.0314	-0.0799	0.0803	-0.1821*	0.0704
	(0.2593)	(0.3159)	(-2.8913)	(0.1894)	(-0.8107)	(0.6177)	(-1.8407)	(0.5745)
**FDISP**			-0.0444*	0.0758**				
			(-1.8060)	(2.2563)				
**L.disesg**					-0.0168**	0.0176*		
					(-2.3494)	(1.7038)		
**disesg**							-0.0400***	0.0188*
							(-5.3712)	(1.7554)
**_cons**	-2.9341***	-1.3343**	-5.0924***	-4.4317***	-4.6266***	-3.8872***	-5.0642***	-6.1549***
	(-7.2129)	(-2.5595)	(-9.5654)	(-7.5487)	(-12.6933)	(-8.0145)	(-13.6334)	(-13.3744)
***N***	21755	21681	8122	8105	21037	20928	21665	21539
**r2_a**	0.8754	0.5355	0.7581	0.5472	0.8257	0.5377	0.8216	0.5905

Notes:This table provides a robustness test for the relationshipbetween ESG rating divergence and green innovation in enterprises

***, ** and * indicate significance at the 1, 5 and 10% levels, respectively.

**Table 5:** Robustness testing based on probit model, instrumental variable method, and PSM.

	(1)	(2)	(3)	(4)	(5)	(6)
	GPcitationdumm	GPtotaldumm	GPcitation	GPtotal	GPcitation	GPtotal
**disesg**	-0.0398***	-0.0949***	2.5072***	1.5959***	-0.0252***	0.0348***
	(-3.1447)	(-7.3651)	(22.0981)	(19.9708)	(-3.2037)	(2.9744)
**Size**	0.4091***	0.3510***	0.6488***	0.4831***	0.1841***	0.1879***
	(47.1125)	(42.8416)	(41.6856)	(39.7324)	(10.6260)	(8.5101)
**REC**	2.4986***	2.0510***	1.3096***	0.9011***	0.1894	0.9172***
	(27.4100)	(23.7868)	(8.5045)	(8.0770)	(1.4682)	(5.3544)
**SOE**	0.0166	0.1745***	0.2898***	0.3638***	0.0337	0.1471**
	(0.7448)	(8.1468)	(8.5214)	(14.2054)	(0.7274)	(2.5486)
**ListAge**	0.1086***	-0.1200***	0.0549***	-0.1229***	0.2713***	0.0950***
	(7.7013)	(-9.2845)	(2.6301)	(-8.2805)	(10.3114)	(3.0793)
**Growth**	-0.0028	-0.0011**	0.0000	0.0000	0.0000**	-0.0004**
	(-1.0405)	(-2.1527)	(0.2197)	(0.1711)	(2.0658)	(-2.1928)
**Top5**	-1.0499***	-0.4334***	-1.3975***	-0.6098***	-0.0101	0.0685
	(-15.2845)	(-6.6810)	(-13.4740)	(-8.1173)	(-0.1010)	(0.5435)
**_cons**	-10.0921***	-8.3369***	-19.6861***	-13.9748***	-4.5831***	-4.1853***
	(-52.8594)	(-46.7510)	(-35.7759)	(-34.1528)	(-12.3552)	(-8.9078)
***N***	26699	26566	26699	26566	19051	18909
**r2_a**	0.1562	0.1056	0.0616	0.0656	0.8059	0.5295

Notes:This table provides a robustness test for the relationship between ESG rating divergence and green innovation in enterprises

***, ** and * indicate significance at the 1, 5 and 10% levels, respectively.

#### (2) Instrumental variable method

This study uses the Wang et al. [52] method to further remove the impact of endogeneity on the research outcomes, and it chooses the instrumental variable as the average value of ESG rating disagreement in each industry. Due to the information spillover effect, the ESG performance of a single company has an economic impact on the entire sector. As a result, there is a high correlation between the industry’s ESG rating divergence and the enterprise-level ESG rating divergence. The regression results meet the premise of instrumental variable exclusivity since they are consistent with the benchmark regression results (as indicated in Columns 3–4 of Table 5) and the industry average discrepancy has no discernible effect on corporate green innovation.

#### (3) Psm test

The traditional fitting regression will lead to biased estimation results due to selectivity bias and mixed bias. The specific empirical idea is as follows: on the basis of the dummy variable of ESG rating disagreement calculated above, the kernel matching method is adopted, and the control variables such as enterprise size and enterprise age are used as covariates for matching, so that there is no systematic difference between the treatment group and the control group, and the net effect of ESG rating disagreement on corporate green innovation activities is finally obtained,and Table 5’s columns 5–6 display the outcomes.

### 4.4 Effect test of internal strategy and external pressure mechanism

The above has verified the empirical evidence of ESG rating divergence on enterprises’ green innovation activities. Referring to the research of Jiang Ting [53], the following models are used to test the mechanism:

$$M_{it}=disesg_{it}+Size_{it}+REC_{it}+SOE_{it}+ListAge_{it}+Growth_{it}+Top5_{it}+\gamma +\lambda +\delta +\varepsilon_{it}$$ (3)

Among them, *M*_*it*_ is the mechanism variable that measures internal strategic adjustment channels and external pressure channels,the other variables are the same as those found in the benchmark regression model.

On the one hand, Disagreement about an ESG rating, as a negative signal, will, lessen the information’s worth. Because of the short-board impact, managers must modify the company’s strategy to get a higher overall score and lessen disagreement in the upcoming rating cycle. In order to investigate the mechanism by which company strategy influences the relationship between disagreements in ESG ratings and green innovation activities, this article employs R&D investment (RD), capital renewal speed (Dep), and financialization level (finratio) as proxy variables of corporate strategy.

On the other hand, the enterprise governance efficiency, social reputation, and other variables that are closely linked to the firm business prospects and default risk are reflected in the ESG grade. Higher ratings will therefore cut the cost of debt capital, whereas poorer or inconsistent ratings can lead to capital market stock price swings that will affect stock liquidity. Second, analysts will become aware of the rating dispute when word gets out. The information on ESG ratings is professional in and of itself, but disagreements add to the complexity of the data. Analysts can be highly skilled at interpreting ESG data since they work as a professional middleman in the information transmission process between investors and businesses. Investor decision-making is guided by the analysis’s findings, and the enterprises that the analysts are focusing on are capable of professionally interpreting the ESG rating divergence, mitigating the impact of the divergence’s stock price fluctuations, and securing more equity financing and investment in their innovative endeavors. Lastly, the news media can easily report on the rating disparity of listed businesses as a trending issue, drawing the interest of pertinent government agencies and financial authorities. Therefore, in order to examine the external effect mechanism between ESG rating divergence and green innovation activities, this article chooses to study the cost of equity capital (Cec), analyst attention (AAttention), and media attention (newsnum) [54] as the proxy variables of external pressure. Table 6 presents the empirical results.

**Table 6:** Mechanism test results of the relationship between internal strategy(RD、Dep、finratio) and external pressure(Cec、AAttention、Newsnum) on ESG rating divergence and green innovation activities of enterprises.

	(1)	(2)	(3)	(4)	(5)	(6)
	Internal strategic channels			External pressure channels		
	RD	Dep	finratio	Cec	AAttention	Newsnum
**disesg**	-0.0190*	-7.1e+07***	-0.2631*	-0.0075*	0.5383***	-24.6015***
	(-1.8628)	(-5.4539)	(-1.7806)	(-1.8843)	(10.7880)	(-4.7185)
**Size**	0.0111	2.3e+08***	0.1112	-0.0124	0.0223	111.8198***
	(0.3894)	(7.9490)	(0.3958)	(-1.5326)	(0.4578)	(11.4092)
**REC**	0.2372	-1.0e+08	-4.2988**	0.0792	-0.6321**	48.6917
	(1.1964)	(-1.1054)	(-2.4948)	(1.6175)	(-1.9890)	(0.8146)
**SOE**	-0.0105	-2.9e+07	0.2793	-0.0011	0.1460	19.0542
	(-0.2080)	(-1.3091)	(1.2845)	(-0.1116)	(1.3935)	(0.9177)
**ListAge**	0.0569	-6.6e+06	0.5708	0.0102	-0.3033***	-47.7855***
	(1.0337)	(-0.1252)	(1.2721)	(1.0447)	(-3.3601)	(-2.7266)
**Growth**	0.0016	1.9e+04*	0.0000	-0.0000	0.0001***	-0.0420***
	(0.3091)	(1.9091)	(0.6184)	(-1.3938)	(7.1311)	(-4.9347)
**Top5**	-0.2014	4.2e+08***	1.5801	0.0329	-0.5659**	-71.7204
	(-1.3085)	(3.0046)	(0.4006)	(0.7553)	(-2.0751)	(-0.7974)
**_cons**	4.3403***	-5.1e+09***	-2.8002	0.3646**	0.0367	-2.2e+03***
	(6.1750)	(-10.0559)	(-0.4380)	(2.0048)	(0.0345)	(-9.2452)
***N***	5655	21597	21565	6230	2747	26215
**r2_a**	0.9134	0.9257	0.0012	0.2451	0.7154	0.7782

Notes:This table provides a channel mechanism test for the relationship between between ESG rating divergence and green innovation in enterprises

***, ** and * indicate significance at the 1, 5 and 10% levels, respectively.

According to the above regression results, operators will change the enterprise’s internal strategy by increasing R&D investment, speeding up asset turnover, and lowering financialization levels in response to agents’ pressure when there is disagreement over ESG ratings. This will have an impact on the enterprise’s green innovation initiatives. Second, external investors need to raise the risk premium brought on by divergence and raise the cost of equity financing for businesses as a result of the information spillover process related to ESG ratings. Furthermore, the dispute will draw the interest of analysts and outside media, which could influence corporate green innovation initiatives through the external environmental supervision channel. The second hypothesis is true.

### 4.5. Moderating effect

#### (1) ESG rating divergence, managers’ self-interest and green innovation activities

According to the principal-agent theory, managers’ self-interested actions to satisfy their own needs for maximization result from the discrepancy between interest demands and information asymmetry, which creates a conflict of interest between shareholders and managers [55]. Owners will search for a variety of assessment techniques to control managers’ self-serving actions and enhance operator supervision. As soon as there is ESG uncertainty, investors will focus more on the lower score and disregard the higher score because of the short board impact. In order to meet the client’s target in the upcoming assessment period, management will typically, when under pressure, manipulate information disclosure, re-integrate and allocate internal resources, and raise the cost of information interpretation. They will also direct the market to reach a decision that is in the company’s and its own best interests. In particular, the management excels in regulating the frequency and content of information disclosure through external channels in order to appease investors and lessen the financial limitations on corporate innovation initiatives. During the internal resource integration stage, they will invest in innovation projects with short cycles and quick results instead of high-quality innovation activities with more investment value in order to fit the ESG rating scoring cycle. Ultimately, they will reduce ESG differences to meet their needs for promotion and assessment. The moderating variable is significantly positive, according to test results in Table 7’s columns 1 and 2. This means that managers’ self-interest exacerbates the asymmetric impact of ESG rating disagreement on businesses’ efforts to innovate sustainably; in other words, formalistic behavior by businesses that prioritize quantity over quality improvement in their pursuit of green innovation is more serious.

**Table 7:** The moderating effect of managerial self-interest(Overpay1) and government quality(GQ) on ESG rating divergence and the relationship between green innovation activities in enterprises.

	(1)	(2)	(3)	(4)
	GPcitation	GPtotal	GPcitation	GPtotal
**disesg**	-0.0179***	0.0215**	-0.0926***	-0.0798*
	(-2.8553)	(2.4607)	(-3.4026)	(-1.8737)
**disesgOverpay1**	0.0212**	0.0278**		
	(1.9985)	(2.0469)		
**Overpay1**	-0.0331	-0.0603		
	(-1.0924)	(-1.5668)		
**disesgGQ**			0.0086***	0.0138***
			(2.6700)	(2.7780)
**GQ**			-0.0270**	-0.0445**
			(-1.9632)	(-2.1665)
**Size**	0.1994***	0.1816***	0.1893***	0.1968***
	(12.5667)	(9.5003)	(12.7296)	(10.6557)
**REC**	0.1326	0.8041***	0.1091	0.8764***
	(1.1813)	(5.4566)	(1.0074)	(6.0259)
**SOE**	0.0964***	0.1652***	0.0633*	0.1759***
	(2.5835)	(3.5113)	(1.6734)	(3.7671)
**ListAge**	0.2384***	0.1308***	0.2604***	0.1091***
	(10.9066)	(5.0003)	(10.9930)	(4.0019)
**Growth**	-0.0011**	0.0000	0.0000***	0.0000
	(-2.2242)	(0.5205)	(3.0254)	(0.5217)
**Top5**	-0.0763	0.0589	-0.0659	0.0257
	(-0.8825)	(0.5414)	(-0.7703)	(0.2385)
**_cons**	-4.8554***	-4.0542***	-4.5098***	-4.0149***
	(-14.3458)	(-9.9626)	(-13.5344)	(-9.4121)
***N***	24272	24447	25953	25822
**r2_a**	0.7942	0.4896	0.8132	0.5376

Notes:This table provides a moderating effect test on the relationship between ESG rating divergence and green innovation in enterprises

***, ** and * indicate significance at the 1, 5 and 10% levels, respectively.

#### (2) ESG divergence, government quality and green innovation activities

Government quality is an important indicator reflecting the level of local government governance, which is mainly reflected in the provision of public services, the construction of ecological and livable environment, the improvement of legal system and the tax and fee system. In the face of ESG rating divergence, enterprises have strong external financing constraints and short-sighted internal managers. In addition, the implementation of local financial system reform and the development of financial markets are important initiatives of high-quality government, and these measures help to improve the transparency and efficiency of the market. High-quality governments can also strengthen the supervision role of external ESG rating soft supervision through digital supervision and legal system regulation, strengthen the supervision of managers’ self-interested behaviors, enhance the trust between entrusted agents, and promote the efficiency and quality of green production of enterprises. Under the guidance of the goal of local GDP growth and fiscal revenue maximization, low-quality governments will have strong motivation to collicit with local enterprises. The local economy also grows rapidly in the short term, thus contributing to the myopic effect of innovation. To sum up, the difference in the quality of local governments will significantly affect the intensity of information orientation and cost orientation of ESG ratings, thus having an impact on corporate green innovation. The empirical test results are shown in columns 3–4 of Table 7. Through the test results, it can be seen that the moderating variables are significantly negative in both the quantity and quality dimensions of green innovation.

## 5.Further analysis

### 5.1 Heterogeneity analysis

#### (1) Heterogeneity of environmental regulation

Environmental regulations can serve as an effective tool for strict oversight, increasing the penalties meted out to local governments for environmental infractions. They can also compel businesses to make decisions based on sustainable development and the environment, as well as to modify and improve their corporate plans. ESG, on the other hand, supports environmental legislation by acting as a soft restriction. As a gauge of corporate sustainability, the ESG rating will have less legal weight in areas with laxer environmental laws. Businesses do not prioritize their sustainable strategy transformation, even when they are aware that their actions run counter to the ESG idea. However, ESG is a crucial evaluation criterion for the extent to which the enterprise’s sustainable idea is implemented in areas with stringent environmental requirements. When an enterprise receives a different ESG rating, not only will investors and other external stakeholders be wary, but environmental protection supervision and enforcement departments will also keep an eye on it and conduct investigations. This will force the enterprise to refrain from allocating resources to green investments in order to avoid facing applicable administrative penalties and thereby raise the bar for green innovation. Referring to the research of Yin Lihui and Wu Chuan-qing [56], we calculate the word frequency of environmental protection in the government work report of each province as the proxy variable to measure the environmental regulation intensity of each province. This allows us to investigate whether ESG divergence has heterogeneity on enterprise green innovation activities in regions with different environmental regulation intensity. The regions with stronger environmental regulation, which is set at 1, are the provinces that have stronger environmental regulations than the average. The test results in Table 8 show that in regions with weaker environmental regulations than in those with stronger regulations, there is a greater beneficial impact of ESG rating divergence on corporate green quantitative innovation. Nonetheless, it exhibits a substantial encouraging effect in areas with strict environmental regulations. The explanation is that enterprise managers engage in less substantive and more formalistic innovation in areas with lax environmental regulations. Businesses in areas with strict restrictions must pay hefty fines for environmental infractions.

**Table 8:** Results of environmental regulation heterogeneity test.

	(1)	(2)	(3)	(4)
	Weak environmental regulations		Strong environmental regulations	
	GPcitation	GPtotal	GPcitation	GPtotal
**disesg**	-0.0214**	0.0483***	0.0208**	0.0279**
	(-1.9751)	(3.1247)	(2.1325)	(2.0282)
**Size**	0.1563***	0.2387***	0.2169***	0.1380***
	(6.7483)	(8.5820)	(9.3661)	(4.4592)
**REC**	0.0103	1.2273***	0.1383	0.6474***
	(0.0514)	(4.8283)	(0.9574)	(3.1585)
**SOE**	0.1483**	0.1829**	-0.0521	0.1947***
	(2.2936)	(2.2853)	(-1.0586)	(3.0665)
**ListAge**	0.2187***	0.1083**	0.2889***	0.1270***
	(5.7680)	(2.5484)	(8.7516)	(2.8753)
**Growth**	-0.0004***	-0.0005***	0.0001***	0.0000
	(-3.7118)	(-3.8549)	(5.7124)	(1.2350)
**Top5**	0.1069	0.3042*	-0.1793	-0.2731
	(0.7616)	(1.8364)	(-1.4281)	(-1.6006)
**_cons**	-4.0735***	-5.4756***	-5.3232***	-2.7422***
	(-8.1376)	(-8.9372)	(-10.7074)	(-4.0973)
***N***	10972	11519	12484	12925
**r2_a**	0.7880	0.5370	0.8319	0.5574

Notes:This table provides the Environmental Regulation heterogeneity test of the relationship between ESG rating divergence and green innovation in enterprises

***, ** and * indicate significance at the 1, 5 and 10% levels, respectively.

#### (2) Heterogeneity of pollution degree

The research samples are classified as heavy polluting samples with 1 and non-heavy polluting enterprises with 0 based on the heavy polluting industries as defined in the Guidelines for Environmental Information Disclosure of Listed Companies published by the Ministry of Environmental Protection in September 2010. The split was necessary because, in contrast to other businesses, the division is paying increasing attention to the rights and interests of employees, corporate ethics, and other factors, in addition to drawing social attention owing to environmental degradation. In addition to stakeholders and outside monitors, ESG grading agencies have particular preferences when it comes to pollution levels. As a result, heavy polluting businesses are more susceptible to sustainable development metrics like the ESG rating than are non-heavy polluting businesses. ESG ratings are split under the short board effect. Table 9 demonstrates that the disagreement in ESG ratings significantly promotes high-quality green innovation activities in highly polluting enterprises. Additionally, the quantitative impact on green innovation shows a downward trend in the significance coefficient due to the crowding-out effect of invested funds. Non-polluting businesses, on the other hand, are less sensitive to the ESG rating, so the management is less likely to notice divergence and to modify the current strategy to align with the ESG rating concept. Instead, they will likely stick to the original innovation strategy of prioritizing quantity over quality in order to satisfy clients and external investors. As a result, when there is a divergence in the ESG rating, businesses that pollute a lot will be more aware of it and will act fast to find coping mechanisms to find high-quality green innovation development, while other businesses will continue to exhibit the formal characteristics of innovation for ESG rating divergence.

**Table 9:** Heterogeneity test results of pollution levels.

	(1)	(2)	(3)	(4)
	Non heavily polluting enterprises		Heavy polluting enterprises	
	GPcitation	GPtotal	GPcitation	GPtotal
**disesg**	-0.0251***	0.0312***	0.0258**	0.0301*
	(-3.4449)	(3.0681)	(2.1110)	(1.6513)
**Size**	0.1848***	0.1664***	0.2244***	0.2143***
	(10.5242)	(7.6751)	(7.1581)	(5.1054)
**REC**	0.1967	0.9434***	-0.4414*	0.2848
	(1.6399)	(5.8326)	(-1.6908)	(0.7324)
**SOE**	0.0965**	0.1684***	-0.0644	0.1686
	(2.3031)	(3.1904)	(-0.8563)	(1.6112)
**ListAge**	0.2859***	0.1006***	0.1675***	0.2293***
	(11.2072)	(3.4116)	(3.7905)	(4.2208)
**Growth**	0.0000***	0.0000	-0.0116**	0.0066
	(3.1319)	(0.7759)	(-2.0548)	(1.3460)
**Top5**	-0.0775	0.0547	-0.1684	-0.0885
	(-0.7801)	(0.4367)	(-1.0582)	(-0.3938)
**_cons**	-4.6161***	-3.7685***	-5.3405***	-4.5978***
	(-12.2573)	(-8.1227)	(-7.9483)	(-5.0280)
***N***	20103	19988	5960	6184
**r2_a**	0.8200	0.5460	0.7936	0.5262

Notes:This table provides the Pollution Levels heterogeneity test of the relationship between ESG rating divergence and green innovation in enterprises

***, ** and * indicate significance at the 1, 5 and 10% levels, respectively.

#### (3) Heterogeneity of enterprise size

Large investment, significant risk, and a lengthy cycle are characteristics of enterprise innovation. Furthermore, the enterprise size is determined by enterprise assets; enterprises with less than the average asset size are defined as small enterprises, with a value of 0, and enterprises with more than the average asset size are defined as large enterprises, with a value of 1. This helps investigate whether the impact of the short board effect will be heterogeneous due to different enterprise sizes when ESG ratings differ. Table 10 presents empirical evidence indicating that disagreements in ESG ratings have a detrimental impact on the quality of green innovation but a negligible stimulating effect on the quantity of green innovation produced by small-scale firms. Due to their severe financial limitations, small businesses find it challenging to commit their meager resources to high-risk, long-term green innovation projects—especially those of superior quality. In contrast, large-scale businesses are better equipped to identify and respond to hazards, have more consistent funding sources, and are resilient to both the risks associated with innovation and the unpredictability of the outside world. Large-scale companies are more likely to invest more in innovation in order to pursue financial gains and to appease stakeholders when their ESG ratings diverge. Consequently, there is a greater stimulant effect of ESG rating disparity on large-scale firms’ green innovation initiatives.

**Table 10:** Results of heterogeneity test for enterprise scale.

	(1)	(2)	(3)	(4)
	Small scale enterprises		large-scale enterprises	
	GPcitation	GPtotal	GPcitation	GPtotal
**disesg**	-0.0135**	0.0073	0.0351*	0.0687**
	(-2.2527)	(0.8698)	(1.7424)	(2.2335)
**Size**	0.1846***	0.1835***	0.1145***	0.3125***
	(11.3140)	(8.7389)	(2.5812)	(5.3225)
**REC**	0.0176	0.6738***	0.5673*	1.9417***
	(0.1643)	(4.6236)	(1.8446)	(3.8229)
**SOE**	0.0310	0.1244***	-0.1039	0.4017**
	(0.7973)	(2.6626)	(-1.0589)	(2.5204)
**ListAge**	0.2489***	0.0419*	0.6284***	-0.1272
	(11.8619)	(1.6544)	(8.7386)	(-1.2571)
**Growth**	-0.0007**	-0.0004	-0.0022**	0.0000
	(-2.3324)	(-1.3772)	(-2.2591)	(0.4778)
**Top5**	-0.2740***	0.0602	-0.1296	0.3621
	(-2.8780)	(0.5168)	(-0.6466)	(1.2815)
**_cons**	-4.1233***	-3.8731***	-4.1629***	-7.1895***
	(-11.9034)	(-8.6703)	(-3.9393)	(-5.1184)
***N***	20019	19968	5941	6042
**r2_a**	0.7897	0.4474	0.8608	0.6244

Notes:This table provides the Enterprise Scale heterogeneity test of the relationship between ESG rating divergence and green innovation in enterprises

***, ** and * indicate significance at the 1, 5 and 10% levels, respectively.

### 5.2 Adjustment of the short board impact

The short board effect of rating divergence is mostly due to the significant ESG rating difference brought on by the limited accessibility of enterprise information both internally and externally. In particular, inadequate information sharing amongst the trusted agents within the company exacerbates the agency cost issue by forcing managers and shareholders to handle things on their own. In this instance, firms’ external information sharing is deficient, creating more room for ESG rating organizations to independently interpret the data, which exacerbates already existing disparities. Consequently, the short-board effect of the ESG rating can be corrected by unblocking the enterprise’s internal information sharing and external information circulation mechanism and lowering the internal and external information asymmetry.

The present study used the quantitative approach of digital transformation (Indigital), as suggested by Yuan et al. [57], to gauge the accessibility of internal information. The information disclosed by businesses tends to be consistent because in this way, an effective communication mechanism can be formed within the organization, communication barriers between departments can be reduced, decisions made based on managers’ preferences can be further avoided, and inefficient production, investment, and communication can be greatly reduced due to information asymmetry. Based on this, disclosure (Disclosure) is employed as the proxy variable to measure how accessible external information is, with reference to Wu et al.’s research [58]. In Table 11, the test results are displayed in columns 1–2.

**Table 11:** The industry(mean_x) and spatial(mean_y) impacts of ESG rating divergence.

	(1)	(2)	(3)	(4)
	disesg	disesg	mean_x	mean_y
**Disclosure**	0.0212**			
	(2.0983)			
**Size**	-0.0812***	-0.0833***	0.0034	0.0034
	(-4.6798)	(-6.7251)	(0.8883)	(0.8883)
**REC**	-0.0078	0.1635	0.0260	0.0260
	(-0.0665)	(1.6252)	(0.9419)	(0.9419)
**SOE**	-0.1023***	-0.0758***	-0.0251***	-0.0251***
	(-3.0290)	(-2.6088)	(-3.2246)	(-3.2246)
**ListAge**	-0.0112	0.2670***	0.0246***	0.0246***
	(-0.2918)	(8.9418)	(3.8910)	(3.8910)
**Growth**	-0.0000	-0.0000	0.0000	0.0000
	(-1.5819)	(-0.8741)	(1.2827)	(1.2827)
**Top5**	0.2036*	0.5438***	-0.0303	-0.0303
	(1.9279)	(7.0312)	(-1.4119)	(-1.4119)
**Indigital**		0.0002***		
		(4.0595)		
**disesg**			0.0165***	0.0165***
			(8.0315)	(8.0315)
**_cons**	3.6703***	3.3001***	1.8968***	1.8968***
	(10.1840)	(12.8054)	(23.8145)	(23.8145)
***N***	17382	25559	26196	26196
**r2_a**	0.4104	0.3849	0.7839	0.7839

Notes:This table provides the results of correcting the weakness effect and testing the contagion mechanism

***, ** and * indicate significance at the 1, 5 and 10% levels, respectively.

### 5.3 Divergence in ESG ratings and its effects on industry and geography

Because ESG rating agencies consider enterprise, industry, and geographic area variables while conducting ratings, there is an external contagion impact associated with ESG rating divergence. As an illustration, when it comes to the carbon emission dimension, the coking coal industry’s carbon emission standard line differs from the finance industry’s, even though it is the same for both. Or, in terms of creating jobs, every province has a different demographic base, and every business will take on a different number of positions. For this reason, rating agencies must develop ESG grading standards based on regional or industrial features. Because same rating standards apply to businesses in similar geographic locations, the difference will be similar. Consequently, the ESG rating’s divergence degree spreads to businesses in nearby industries or spaces. Thus, the mean value of other businesses in the same industry, excluding the business’s own rating disagreement, is calculated in this paper as the proxy variable of the business’s industrial contagion effect (mean_x), and the mean value of other businesses in the same province, excluding the business’s own rating disagreement, is calculated as the proxy variable of the business’s regional contagion effect (mean_y). In Table 11, the test results are displayed in columns 3–4.

## 6. Concluding remarks and advice

China is still in the early stages of developing its ESG practices, and the lack of uniformity in standards and information has made ESG rating findings unclear. This has drawn interest from a wide range of sources. In this regard, this research empirically analyzes the mechanism of ESG rating divergence’s influence on corporate green innovation activities using sample data of listed businesses from 2009 to 2022. Its primary findings are as follows. Second, through both the external pressure channel and the internal strategy adjustment channel, ESG rating disparity affects company green innovation initiatives. Third, while the quality of government can lessen this imbalance, managers’ self-interest exacerbates the difference in the ESG ratings for business green innovation initiatives. Fourthly, ESG rating differential encourages high-quality green innovation activities while lowering the quantity of green innovation that accumulates in large-scale, non-heavy polluting, and places with strong environmental regulations. Fifth, the short board effect of ESG rating divergence can be substantially mitigated by advancing the digital transformation of organizations and improving the quality of enterprise information disclosure. ESG rating divergence has a contagion effect across industries and geographies.

The research conclusion of this article indicates that ESG rating divergence has asymmetric effects on corporate innovation activities. And the impact mechanism of the two was deeply explored, providing a new research perspective for the diversified impact of ESG rating results. However, this article does not explain the temporal characteristics of ESG rating divergence. As people’s understanding of ESG deepens, further exploration is needed to determine whether their understanding of ESG ratings will gradually become consistent. And the economic consequences of ESG rating differences on corporate innovation activities have not been fully explained yet.This is a further research direction in the future.

Drawing from the aforementioned conclusions, this study proposes the following recommendations:

1. Businesses must raise the bar for green innovation and disclose external information more effectively. In addition to being necessary for the new development concept, green innovation is a practical solution for both environmental preservation and the economic conundrum. Therefore, in order to get a competitive advantage in the highly competitive market, businesses need allocate greater resources to green innovation initiatives. Businesses should also increase the amount of ESG information disclosed, lessen the discrepancy in rating outcomes brought on by external rating agencies’ information asymmetry, mitigate the cognitive bias of investors in the market regarding information, lessen managers’ self-serving behavior, expedite the dissemination and transparency of corporate information, and guarantee that external rating agencies are able to fairly and accurately assess and judge corporate actions.
2. The ratings issued by ESG rating firms act as soft market limitations, directing capital market investment choices. As such, it is imperative to guarantee the impartiality and equity of rating agencies. On the one hand, we ought to closely monitor the pertinent data that businesses, particularly those located in areas with a high level of environmental control, divulge, and develop a unique rating system just for them. On the other hand, it’s critical to correctly pinpoint the self-serving actions of managers and the shortsightedness of businesses, guarantee the validity and correctness of rating outcomes, and give capital market participants more precise and objective information.
3. The creation of an ESG information disclosure system must to be vigorously encouraged by the government and pertinent supervisory bodies. The comprehension of ESG will progressively become more cohesive as it continues to grow. To provide the proper institutional assurance for the efficient operation of China’s securities market and excellent economic development, improve the information disclosure system, raise the cost of illegal information disclosure, and standardize the rating standard.

## Supporting information

S1 Text (DO)

S2 Text (DO)

S3 Text (DTA)

S4 Text (DTA)
